# Consensus clustering and functional interpretation of gene-expression data

**DOI:** 10.1186/gb-2004-5-11-r94

**Published:** 2004-11-01

**Authors:** Stephen Swift, Allan Tucker, Veronica Vinciotti, Nigel Martin, Christine Orengo, Xiaohui Liu, Paul Kellam

**Affiliations:** 1 Department of Information Systems and Computing, Brunel University, Uxbridge UB8 3PH, UK; 2 School of Computer Science and Information Systems, Birkbeck College, London WC1E 7HX, UK; 3 Department of Biochemistry and Molecular Biology, University College London, London WC1E 6BT, UK; 4 Virus Genomics and Bioinformatics Group, Department of Infection, Windeyer Institute, 46 Cleveland Street, University College London, London W1T 4JF, UK

## Abstract

Microarray analysis using clustering algorithms can suffer from lack of inter-method consistency in assigning related gene-expression profiles to clusters. Obtaining a consensus set of clusters from a number of clustering methods should improve confidence in gene-expression analysis. Here we introduce consensus clustering, which provides such an advantage. When coupled with a statistically based gene functional analysis, our method allowed the identification of novel genes regulated by NFκB and the unfolded protein response in certain B-cell lymphomas.

## Abstract 2

Consensus clustering, a new method for analyzing microarray data that takes a consensus set of clusters from various algorithms, is shown to perform better than individual methods alone.

## Background

There are many practical applications that involve the grouping of a set of objects into a number of mutually exclusive subsets. Methods to achieve the partitioning of objects related by correlation or distance metrics are collectively known as clustering algorithms. Any algorithm that applies a global search for optimal clusters in a given dataset will run in exponential time to the size of problem space, and therefore heuristics are normally required to cope with most real-world clustering problems. This is especially true in microarray analysis, where gene-expression data can contain many thousands of variables. The ability to divide data into groups of genes sharing patterns of coexpression allows more detailed biological insights into global regulation of gene expression and cellular function.

Many different heuristic algorithms are available for clustering. Representative statistical methods include k-means, hierarchical clustering (HC) and partitioning around medoids (PAM) [1-3]. Most algorithms make use of a starting allocation of variables based, for example, on random points in the data space or on the most correlated variables, and which therefore contain an inherent bias in their search space. These methods are also prone to becoming stuck in local maxima during the search. Nevertheless, they have been used for partitioning gene-expression data with notable success [4,5]. Artificial Intelligence (AI) techniques such as genetic algorithms, neural networks and simulated annealing (SA) [6] have also been used to solve the grouping problem, resulting in more general partitioning methods that can be applied to clustering [7,8]. In addition, other clustering methods developed within the bioinformatics community, such as the cluster affinity search technique (CAST), have been applied to gene-expression data analysis [9]. Importantly, all of these methods aim to overcome the biases and local maxima involved during a search but to do this requires fine-tuning of parameters.

Recently, a number of studies have attempted to compare and validate cluster method consistency. Cluster validation can be split into two main procedures: internal validation, involving the use of information contained within the given dataset to assess the validity of the clusters; or external validation, based on assessing cluster results relative to another data source, for example, gene function annotation. Internal validation methods include comparing a number of clustering algorithms based upon a figure of merit (FOM) metric, which rates the predictive power of a clustering arrangement using a leave-one-out technique [10]. This and other metrics for assessing agreement between two data partitions [11,12] readily show the different levels of cluster method disagreement. In addition, when the FOM metric was used with an external cluster validity measure, similar inconsistencies are observed [13].

These method-based differences in cluster partitions have led to a number of studies that produce statistical measures of cluster reliability either for the gene dimension [14,15] or the sample dimension of a gene-expression matrix. For example, the confidence in hierarchical clusters can be calculated by perturbing the data with Gaussian noise and subsequent reclustering of the noisy data [16]. Resampling methods (bagging) have been used to improve the confidence of a single clustering method, namely PAM in [17]. A simple method for comparison between two data partitions, the *weighted-kappa *metric [18], can also be used to assess gene-expression cluster consistency. This metric rates agreement between the classification decisions made by two or more observers. In this case the two observers are the clustering methods. The *weighted-kappa *compares clusters to generate the score within the range -1 (no concordance) to +1 (complete concordance) (Table 1). A high *weighted-kappa *indicates that the two arrangements are similar, while a low value indicates that they are dissimilar. In essence, the *weighted-kappa *metric is analogous to the adjusted Rand index used by others to compare cluster similarity [16,19].

Despite the formal assessment of clustering methods, there remains a practical need to extract reliably clustered genes from a given gene-expression matrix. This could be achieved by capturing the relative merits of the different clustering algorithms and by providing a usable statistical framework for analyzing such clusters. Recently, methods for gene-function prediction using similarities in gene-expression profiles between annotated and uncharacterized genes have been described [20]. To circumvent the problems of clustering algorithm discordance, Wu *et al. *used five different clustering algorithms and a variety of parameter settings on a single gene-expression matrix to construct a database of different gene-expression clusters. From these clusters, statistically significant functions were assigned using existing biological knowledge.

In this paper, we confirm previous work showing gene-expression clustering algorithm discordance using a direct measurement of similarity: the *weighted-kappa *metric. Because of the observed variation between clustering methods, we have developed techniques for combining the results of different clustering algorithms to produce more reliable clusters. A method for clustering gene-expression data using resampling techniques on a single clustering method has been proposed for microarray analysis [19]. In addition, Wu *et al. *showed that clusters that are statistically significant with respect to gene function could be identified within a database of clusters produced from different algorithms [20]. Here we describe a fusion of these two approaches using a 'consensus' strategy to produce both robust and consensus clustering (CC) of gene-expression data and assign statistical significance to these clusters from known gene functions. Our method is different from the approach of Monti *et al.*, in that different clustering algorithms are used rather than perturbing the gene-expression data for a single algorithm [19]. Our method is also distinct from the cluster database approach of Wu *et al *[20]. There, clusters from different algorithms were in effect fused if the consensus view of all algorithms indicated that the gene-expression profiles clustered independently of the method. In the absence of a defined rule base for selecting clustering algorithms, we have implemented clustering methods from the statistical, AI and data-mining communities to prevent 'cluster-method type' biases. When consensus clustering was used with probabilistic measures of cluster membership derived from external validation with gene function annotations, it was possible to accurately and rapidly identify specific transcriptionally co-regulated genes from microarray data of distinct B-cell lymphoma types [21].

## Results

### Cluster method comparison

Initially we assessed cluster method consistency for HC, PAM, SA and CAST using the *weighted-kappa *metric and a synthetic dataset of 2,217 gene-expression profiles over 100 time points that partitioned into 40 known clusters. The *weighted-kappa *values derived from the metric indicate the strength of agreement between two observers (Table 1). To interpret two *weighted-kappa *scores, for example, from two cluster arrangements, the broad categories from Table 1 are used, together with an assessment of relative score differences. If the two scores in question were 0.2 and 0.4, one could say that the former is poor (worse) and the latter is fair (better), but not that one is twice as good as the other. To allow defined clusters to be extracted from the tree structure of HC we used the R statistical package [22] implementation of HC. This implementation uses the CUTTREE method to convert the tree structure into a specified number of clusters.

**Figure 1:**
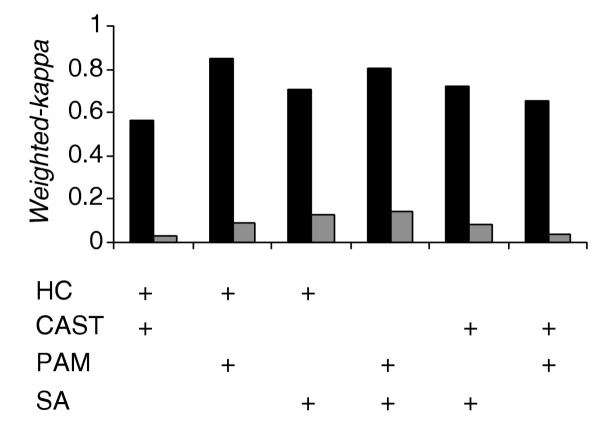
Pairwise comparison of consistency between different cluster algorithm data partitions using the *weighted-kappa *metric (Table 1) to score similarity. Each clustering algorithm was used to analyze the Amersham Score Card dataset (black bars) and the B-cell lymphoma dataset (gray bars), and the cluster-method agreement based on assigning the same genes to the same cluster was calculated and scored. HC, hierarchical clustering; CAST, cluster affinity search technique; PAM, partitioning around medoids; and SA, simulated annealing.

With the synthetic dataset, all clustering algorithms had a 'high' *weighted-kappa *agreement (data not shown) [18]. It is possible that the highly stylized nature of synthetic data resulted in higher than expected cluster-method agreement compared to experimentally derived data. This effect has been observed previously [10,12]. Therefore, we used a repeated microarray control element Amersham Score Card (ASC) dataset as a semi-synthetic validation standard. We also used an experimentally derived microarray dataset for cross-cluster-method comparison. To facilitate cross-method comparison, we used fixed parameters where appropriate (see Materials and methods). Consistent with other studies, we observed that clustering-method consistency varied between methods and datasets (Figure 1). As expected, the repeated gene/probe measurements present in the ASC dataset resulted in higher levels of cluster agreement between methods than the single gene probe B-cell data. With the ASC data there was in general a 'good' level of agreement between all different clustering algorithms, with only CAST compared to HC scoring as 'moderate'. This shows that most clustering methods are able to group highly correlated data accurately, and that repeated measurements of gene-expression values can aid cluster partitioning [12]. Nevertheless, even with such high gene-expression correlation not all cluster assignments were consistent. This effect is magnified with the single probe per gene B-cell lymphoma data, where the degree of agreement for cluster partitioning was less, with no comparison scoring above 'fair'. This observation emphasizes the need for the current desired practice in microarray analysis of using many different clustering algorithms to explore gene-expression data, thereby not over-interpreting clusters on the basis of a single method [23].

### Algorithms

The partial agreement of the different clustering algorithms must reflect the clustering of highly similar gene-expression vectors regardless of the clustering methods used. Where algorithm-based inconsistency problems occur in other aspects of computational biology, such as protein secondary structure prediction, consensus algorithms are often used [24]. These can either report a full or a majority agreement. This consensus strategy has also been applied to explore the effect of perturbing the gene-expression data for a single clustering algorithm [19]. We have therefore designed a similar strategy to identify the consistently clustered gene-expression profiles in microarray datasets by producing a consensus over different clustering methods for a given parameter set (see Materials and methods). Extracting such consistently clustered robust data from a large gene-expression matrix is extremely useful, increasing overall analysis confidence.

#### Robust clustering

**Figure 2:**
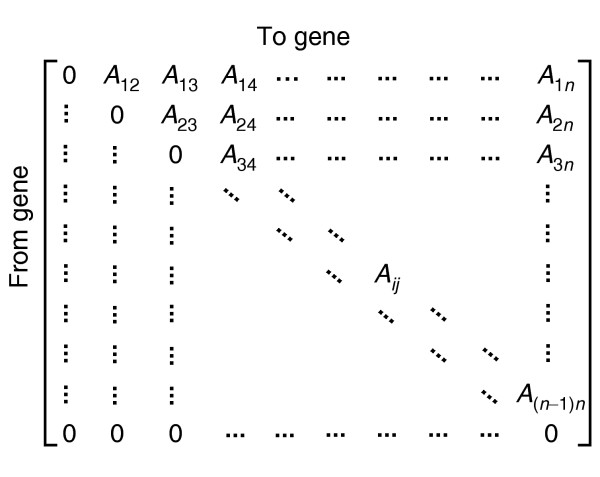
A visual representation of the agreement matrix used as input to robust and consensus clustering. The *n *× *n *matrix is upper triangular. Each cell within the matrix, referenced by column *i *and row *j*, represents the number of clustering methods that have placed gene *i *and gene *j *into the same cluster. In other words, the number represents the agreement between clustering methods concerning gene *i *and gene *j*.

We initially developed an algorithm called robust clustering (RC) for compiling the results of different clustering methods reporting only the co-clustered genes grouped together by all the different algorithms - that is, with maximum agreement across clustering methods. For two genes *i *and *j*, all clustering methods must have allocated them to the same cluster in order for them to be assigned to a robust cluster. This gives a higher level of confidence to the correct assignment of genes appearing within the same cluster. Robust clustering works by first producing an upper triangular *n *× *n *agreement matrix with each matrix cell containing the number of agreements among methods for clustering together the two variables, represented by the row and column indices (Figure 2). This matrix is then used to group variables on the basis of their cluster agreement (present in the matrix).

Robust clustering uses the agreement matrix to generate a list, *List*, which contains all the pairs where the appropriate cell in the agreement matrix contains a value equal to the number of clustering methods being combined (that is, full agreement). Starting with an empty set of robust clusters RC, where *RC*_*i *_is the *i*th robust cluster, the first cluster is created containing the elements of the first pair in *List*. Then the pairs in *List *are iterated through and checked to see if one of the members of the current pair is within any of the existing clusters, *RC*_*i*_.

If one element of the current pair is found and the other element of the pair is not in the same cluster, then the other element is added to that cluster. If neither element of the pair is found in an existing *RC*_*i *_in RC, then a new cluster is added to RC containing each element of the pair. When the end of the list is reached, the set of robust clusters, RC, is the output. The robust clustering algorithm is as follows:

Input:Agreement Matrix (*n *× *n*), *A*

(1) Set *List *= all pairs (*x*, *y*) in the matrix, with agreement = the number of methods

(2) Set *RC *to be an empty list of clusters

(3) Create a cluster and insert the two elements (*x*, *y*) of the first pair in *List *into it

(4) For *i *= 2 to size of *List*-1

(5)  For *j *= 1 to number of Clusters in *RC*

(6)   If *x *or *y *of *List*_*i *_is found within *RC*_*j*_

(7)    If the other member of the pair *List*_*i *_is not found in *RC*_*j*_

(8)     Add the other member to *RC*_*j*_

(9)    End If

(10)   Else If the other member of the pair *List*_*i *_is not found in *RC*_*j*_

(11)    Add a new cluster to *RC *containing *x *and *y*

(12)   End If

(13)  End For

(14) End For

Output:Set of Robust Clusters *RC*

#### Application of robust clustering

Robust clustering was applied to both the ASC and B-cell lymphoma datasets and the partitioning of the gene-expression profiles observed. As expected, the robust clusters do not contain all variables because of the underlying lack of consistent clustering by all methods. As a result, the *weighted-kappa *cannot be calculated. This metric requires both clustering arrangements being compared to be drawn from the same set of items. This is not the case with robust clustering because many items will not be assigned to a cluster. However, approximately 80% of the total ASC data variables and 25% of the B-cell lymphoma variables are assigned to a robust cluster. Robust clustering further subdivides the datasets into smaller clusters, with 24 rather than 13 clusters being defined for ASC, and 154 rather than 40 being defined for the B-cell lymphoma data (Table 2). Robust clusters are therefore valuable for allowing a rapid 'drilling down' in a gene-expression dataset to groups of genes whose coexpression pattern is identified in a manner independent of cluster method.

The robust clustering algorithm is, by definition, subject to discarding gene-expression vectors if only one clustering method performs badly in the co-clustering. This effect of single method under performance on a given dataset has been previously observed for single linkage hierarchical clustering [10,13]. Therefore, to generate clusters with high agreement across methods but not so restrictive as to discard majority consistent variables, we adapted the algorithm to generate consensus clusters, making use of the same agreement matrix.

#### Consensus clustering

Consensus clustering relaxes the full agreement requirement by taking a parameter, 'minimum agreement', which allows different agreement thresholds to be explored. Rather than grouping variables on the basis of full agreement only, consensus clustering maximizes a metric, which rewards variables in the same cluster if they have high cluster method agreement and penalizes variables in the same cluster if they have low agreement. Consensus clustering maximizes agreement using the function *f*(*G*_*i*_) in Equation (1) to score each cluster of size *s*_*i*_



where *A *is the agreement matrix, *G*_*ij *_is the *j*th element of cluster *i *(*G*_*i*_) and *β *is a user-defined parameter (the agreement threshold), which determines whether the score for the cluster is increased or decreased. The score for a clustering arrangement is the sum of the scores of each cluster, which consensus clustering attempts to maximize.

If *β *is equal to *Min*, the minimum value in *A*, then the function is maximized when all variables are placed into the same cluster (that is, a single large cluster). Alternatively, when *β *is equal to *Max*, the maximum value in *A*, the function is maximized when each variable is placed into its own cluster. Essentially all clusters produced by Consensus Clustering are scored by *f*(*G*_*i*_), rewarding and preserving clusters with high agreement between members, while penalizing and discarding clusters containing low agreement between members. A value for *β *should lie between the minimum and the maximum agreement so as not to skew the scoring function. A suitable value for *β *is (*Max *+ *Min*)/2, where *Max *is the maximum value in *A *and *Min *is the minimum. For a uniformly distributed agreement matrix, (*Max *+ *Min*)/2 is the mean value; therefore we penalize values below the mean agreement and reward above it. For both the ASC and B-cell lymphoma data *β *was 2, as *Max *= 4 (four clustering algorithms giving complete agreement) and *Min *= 0 (no agreement). In order to maximize the scoring function for consensus clustering, a search over possible cluster membership is needed. There are many methods for performing a search and it was decided that SA was best because it is an efficient search/optimization procedure that does not suffer from becoming stuck in local maxima. The consensus algorithm is as follows:

Input: Agreement Matrix (*n *× *n*), *A; *Maximum Number of Clusters sought, *m; *Number of Iterations, *Iter; *Agreement Threshold, *β; *Initial Temperature, *θ*_0_*; *Cooling Rate, *c*

(1) Generate a random number of empty clusters (<m)

(2) Randomly distribute the variables (genes) 1..*n *between the clusters

(3) Score each cluster according to Equation (1)

(4) For *i *= 1 to *Iter *do

(5)  Either Split a cluster, Merge two clusters or Move a variable (gene) from one random cluster to another

(6)  Set Δ*f *to difference in score according to Equation (1)

(7)  If Δ*f *< 0 Then

(8)  Calculate probability, *p*, according to Equation (2)

(9)  If *p *> random(0,1) then undo operator

(10) End If

(11)  *θ*_*i *_= *cθ*_*i*-1_

(12) End For

Output:Set of Consensus Clusters

Note that *random*(0,1) (line 9) returns a random uniformly distributed real number between 0 and 1.

The 'split', 'merge' and 'move' operators (line 5) are as follows and used with equal probability:

Split a cluster:

Input: Cluster *g *of size *n*

(1) Randomly shuffle the cluster

(2) Set *i *to be a random whole number between 1 and *n*-1

(3) Create two empty clusters *g*_1 _and *g*_2_

(4) Add elements 1..*i *of *g *to *g*_1_

(5) Add elements i+1..*n *of *g *to *g*_2_

Output:Two new clusters *g*_1 _and *g*_2_

Here the old cluster is deleted and the two new clusters are then added to the set of clusters.

Merge two clusters:

Input: Two Clusters *g*_1 _and *g*_2_

(1) A new cluster *g *is created by forming the union of *g*_1 _and *g*_2_

Output: A new cluster *g*

Here the old clusters are deleted and new cluster is then added to the set of clusters.

Move a gene:

Input:A set of clusters *G*

(1) Two random clusters *g*_1 _and *g*_2 _are chosen where the size of *g*_1 _is greater than one

(2) A random element of *g*_1 _is moved into *g*_2_

Output:The updated set of clusters *G*

The probability (*p*) (line 8) is calculated by:



In the following experiments we found *θ*_0 _= 100, *c *= 0.99994 and *iter *= 1,000,000 as the most efficient parameters for SA.

These parameter settings for SA are effectively determined by the *iter *setting. We denote the change in fitness during the SA algorithm as Δ*f *and the starting temperature as *θ*_0 _which is always positive. From equation 2 it can be clearly seen that if Δ*f *= *θ*_0 _then the (worse) solution will be accepted with probability 0.368 (*e*^-1^). As the temperature cools, this probability will reduce. Here we set *θ*_0 _to be the average of Δ*f *over 1,000 trial evaluations, so that at the beginning of the algorithm, the average worse solution (Δ*f *= *θ*_0_) will be accepted with the probability stated above.

It can be seen from the consensus algorithm that during the *i*th stage of the SA algorithm *θ*_0 _= *θ*_0_*c*^*i*^. The SA algorithm works by assuming that the temperature reduces to zero over an infinite number of iterations. As it is not practical to run the SA algorithm to infinity the method is usually terminated after a fixed number of iterations, (*iter*). At this time the temperature will not be zero, but very small and positive, say *ε*. Therefore,



Hence if some small positive value for *ε *is chosen, and the algorithm is to run for a defined number of iterations (*iter*), then the decay constant *c *is calculated as above.

#### Application of consensus clustering

As consensus clustering relaxes the 'complete agreement' criteria we would expect the majority but not necessarily all robust cluster members to be assigned to the same consensus clusters. This was indeed true for the B-cell data where consensus clustering of the datasets showed that 98.5% of the B-cell robust clusters were assigned correctly to their respective consensus clusters. With the more consistent ASC data 100% of the robust clusters were assigned to the correct consensus clusters.

**Figure 3:**
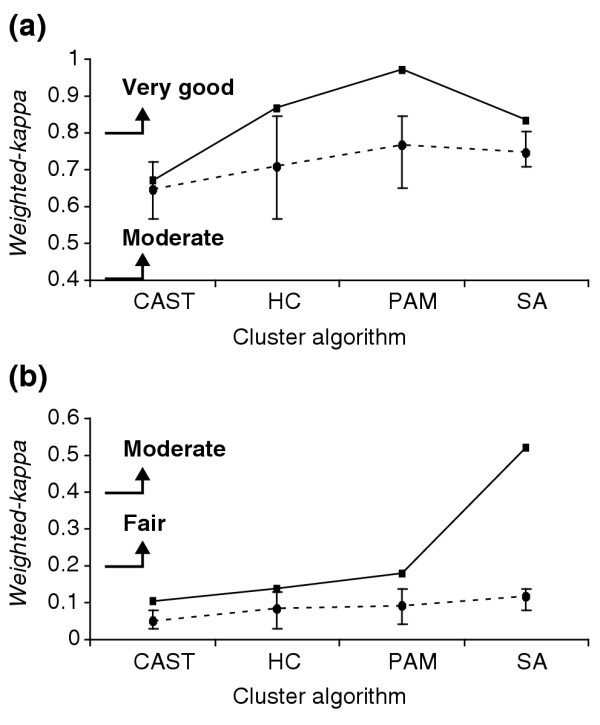
Comparison between consensus clustering and pairwise clustering. The *weighted-kappa *score for consensus clustering (solid line) calculated by comparing consensus clusters to the corresponding individual clustering algorithm is shown relative to mean pairwise *weighted-kappa *score for each single method compared to all other single methods (broken line) for **(a) **the ASC dataset, **(b) **the B-cell lymphoma dataset. The maximum and minimum *weighted-kappa *scores for the collection of single methods are indicated by the error bars. The definitions of *weighted-kappa *scores are derived from Table 1. The parameter settings for the clustering algorithms are: HC and PAM, 13 clusters for the ASC dataset and 40 for the B-cell dataset; CAST, affinity level 0.5; and SA, *θ*_0 _= 100, *c *= 0.99994 and number of iterations = 1,000,000.

The advantage of consensus clustering over all single-cluster methods was evident when comparing consensus clustering to the mean *weighted-kappa *score for each pairwise combination of individual clustering algorithms (derived from Figure 1). Comparisons for the ASC dataset (Figure 3a) and B-cell lymphoma data (Figure 3b) show that consensus clustering improves on all single methods regardless of dataset, except in the case of CAST compared to SA for the ASC dataset (Figure 3a). It is interesting to observe that consensus clustering has higher agreement with SA compared to SA agreement with all other methods in the B-cell data (Figure 3b). The reasons for this are unclear, but suggest that with datasets similar to the B-cell data, SA captures a reliably partitioned subset of the data. To determine if consensus clustering was consistently superior to the use of single clustering methods, particularly the stochastic methods CAST and SA, we performed 10 independent runs of CAST, SA and consensus clustering. From the resulting clusters we determined the mean *weighted-kappa *scores for 45 possible comparisons (that is, the number of unique pairs formed from 10 objects = 10 × 9/2) (Table 3). Consensus clustering provided an extremely high degree of consistency over all 10 runs, with a mean *weighted-kappa *score of 0.96. Importantly, there was little variation between each of the 10 runs with a standard deviation of the mean *weighted-kappa *of 0.0015. SA had a similar low standard deviation, but produced lower inter-run consistency (mean *weighted-kappa *of 0.816). CAST was the least consistent of the methods (mean *weighted-kappa *of 0.646). The differences in the consensus clustering mean compared to SA and CAST are significant at greater than the 99.9% confidence level, thereby showing consensus clustering identifies a reliable data partition, which is significantly better than multiple runs of single clustering methods.

We wished to confirm that the benefit of consensus clustering was not simply due to the parameter settings chosen for the dataset used. This could be confirmed by extensively varying each algorithm's parameter settings and comparing cluster partitioning using the same dataset; however, the large number of combinations of possible parameter settings between all methods makes this unrealistic. An alternative approach is to compare all methods on additional datasets. We therefore tested consensus clustering on two different simulated datasets containing 60 defined clusters of genes. The first synthetic dataset was generated from a vector autoregressive process (VAR) and the second using a multivariate normal distribution (MVN). The number of genes in each cluster varied from 1 to 60, with the number of conditions (arrays) set to 20. The datasets therefore contained 1,830 genes over 20 conditions. As the structure of each dataset is known, the results of each clustering method can be evaluated for accuracy using the *weighted-kappa *metric. Cluster accuracy using the single methods ranged between a *weighted-kappa *of 0.505 to 0.7 (mean *weighted-kappa *of 0.614) (Table 4). It is interesting to note that the single clustering methods performed differently on the two synthetic datasets, with HC, SA and CAST performing better on the MVN synthetic data and PAM better on the VAR synthetic data. Consensus clustering was superior to all single clustering algorithms with *weighted-kappa *scores of 0.725 and 0.729 for VAR and MVN respectively, demonstrating that consensus clustering is accurate regardless of subtleties in the data structure (Table 4).

### Interpretation of consensus clustering

Consensus clustering greatly improves the accuracy of identifying cluster group membership based solely on the gene-expression vector, but as with other clustering algorithms still produces essentially unannotated clusters which require further external validation by gene function analysis. To address this problem, we derived a probability score to test the significance of observing multiple genes with known function in a given cluster against the null hypothesis of this happening by chance. This identifies clusters of high functional group significance, aiding assignment of functions to unclassified genes in the cluster using the 'guilt by association' hypothesis.

The probability score is based on the hypothesis that, if a given cluster, *i*, of size *s*_*i*_, contains *x *genes from a defined functional group of size *k*_*j*_, then the chance of this occurring randomly follows a binomial distribution and is defined by:



where *n *is the number of genes in the dataset. As *k*_*j *_and *x *may potentially be very large, *Pr *from the above equation would be difficult to evaluate. Therefore the normal approximation to the binomial distribution can be used as defined by:



Large positive values of *z *mean that the probability of observing *x *elements from functional group *j *in cluster *i *by chance is very small, (for example *z *> 2.326 corresponds to a probability less than 1%). Note that we perform a one tailed test as we are only interested in the case where a significantly high number of co-clustered genes belong to the same functional group.

**Figure 4:**
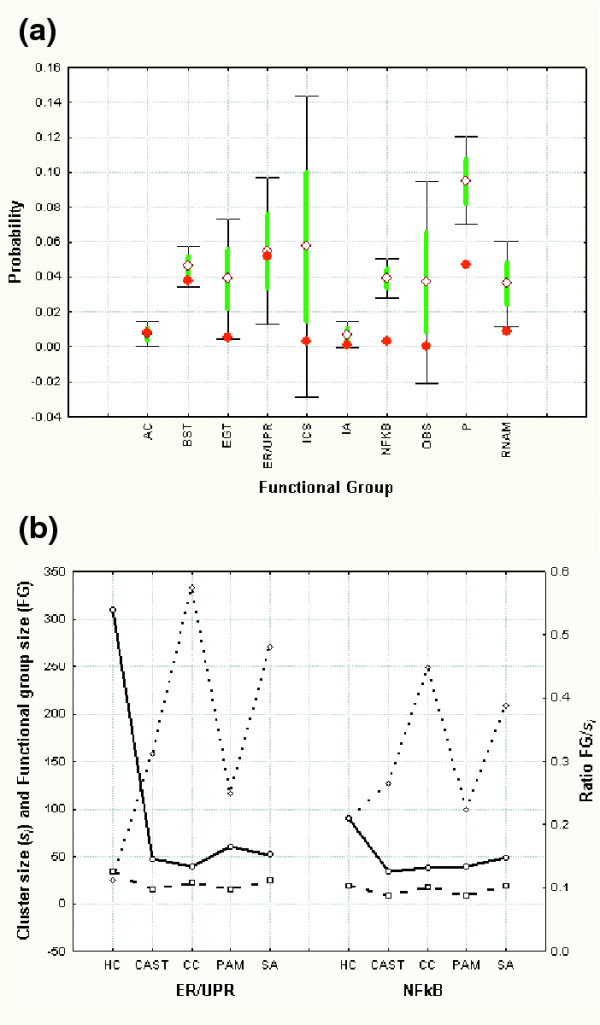
Probability scores and cluster size. **(a) **The lowest probability scores determined for clusters containing the following functional group signature genes were identified: AC, actin cytoskeleton; BST, B-cell signal transduction; EGT, ER/Golgi trafficking; ERUPR, ER stress/unfolded protein response; ICS, immunoglobulin class switching; IA, inflammation and adhesion; NFκB, NFκB signaling; OBS, other B-cell signaling; P, proliferation; RNA, RNA maturation and splicing. The mean (open diamond), standard error (green line) and standard deviation (thin black line and bars) of the minimum probability scores for SA, CAST, HC and PAM are shown together with the minimum probability score for the corresponding consensus cluster (red circle). **(b) **The cluster size (*s*_*i*_) (open circles) and number of defining functional group genes (FG) (open squares) for the NFκB signaling and ER/UPR functional groups are shown together with the FG/*s*_*i *_ratio (open diamonds).

This cluster function probability score was used to identify statistically significant (at the 1% level) B-cell consensus clusters containing defined genes known to be associated with 10 functional groups [21]. To determine if consensus clustering was better able to identify important functional group clusters we determined the functional group probability scores produced by individual clustering algorithms analogous to the strategy of Wu *et al*. [20]. For each functional group, the mean lowest probability scores (using Equation (4)) were calculated for the signal clustering methods and compared to consensus clustering (Figure 4a). Consensus clustering always produced equivalent or lower probabilities for each functional group, indicating that it produced more informative clusters.

One potential confounding factor in this analysis is that consensus clustering achieves a lower probability score by finding smaller clusters. This would decrease the ability to associated new genes with a given functional group. In the worst case the number of genes defining a functional group (FG) would equal the cluster size (*s*_*i*_) (FG/*s*_*i *_= 1). Alternatively, single clustering methods may produce lower probability scores by increasing the cluster size, thereby pulling many genes into the cluster resulting in a FG/*s*_*i *_ratio tending towards zero. This would also reduce the usefulness of the clusters. We determined the cluster size and functional group size for two representative functional groups where the consensus clustering probability was similar to the single method probability score, namely the endoplasmic reticulum (ER) stress response (also known as the unfolded protein response) (ER/UPR) functional group, or the markedly better nuclear factor-κB (NFκB) functional group (Figure 4b). Apart from SA, all single clustering methods tended to produce larger clusters, thereby decreasing the FG/*s*_*i *_ratio. In the most extreme case of the ER/UPR functional group, the HC cluster size was 310 compared to the consensus clustering size of 40. SA tended to produce similar cluster sizes as consensus clustering but with higher overall probabilities. Therefore, consensus clustering identifies significant functional clusters while achieving a workable balance between large or small cluster sizes.

**Figure 5:**
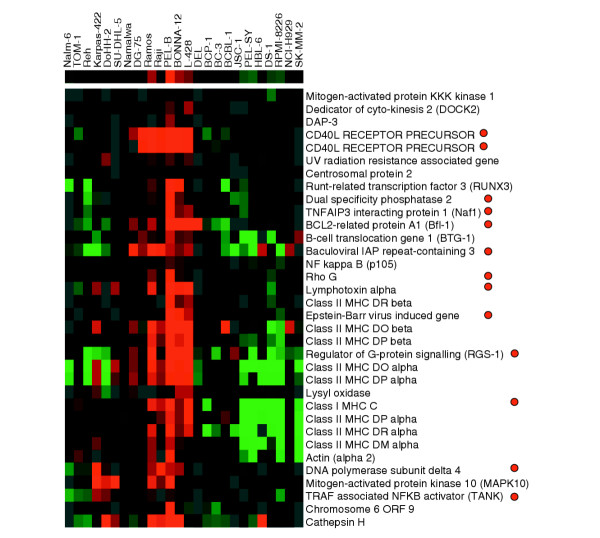
Visualization of average linkage HC using the programs Cluster and Treeview [5] of the NFκB responsive gene cluster identified from consensus clustering and functional annotation. The sample names correspond to different leukemia and lymphoma samples [21], with the NFκB-responsive gene cluster being predominantly expressed in the cell lines Raji, PEL-B, EHEB, BONNA-12 and L-428. Gene names with red circles represent those genes that contain one or more NFκB-binding sites in the region up to 1,000 bp upstream from the putative transcriptional start site.

**Figure 6:**
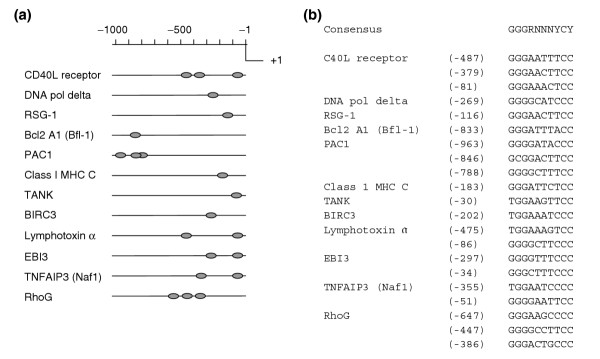
Location and consensus sequence of NFκB-binding sites. **(a) **Position of the NFκB-binding sites identified in the upstream 1,000-bp regions of each gene. The gray ovals represent the position of each binding site shown in **(b) **where the nucleotide sequence for each respective NFκB-binding site is shown relative to the consensus NFκB-binding site GGGRNNNYCY (R is G or A (purine), Y is T or C (pyrimidine) and N is any nucleotide).

We further investigated the two groups NFκB and ER/UPR to assess what additional insights consensus clustering allowed. These two functional groups represent important B-cell functions at different stages of the B-cell development pathway. The consensus cluster associated with NFκB also contained genes either not previously associated with or only tentatively associated with NFκB activity in subsets of B-cell lymphomas. The gene-expression profiles from this consensus cluster were visualized by average linkage HC using the programmes Cluster and Treeview [5] (Figure 5) and clustered gene functions were investigated further using the annotation resources DAVID [25] and GeneCards [26]. From GeneCards each gene was identified in the complete human genome sequence using Ensembl [27] and 1,000 base pairs (bp) upstream of the predicted transcriptional start site extracted for promoter analysis using the program TESS from the Baylor College sequence analysis software BCM [28] (Figure 6).

This consensus cluster is predominantly overexpressed in the cell lines Raji, Pel-B, EHEB, Bonna-12 and L-428. These cell lines have in common the induction of the NFκB pathway, either through expression of Epstein-Barr virus LMP-1 protein (Raji, Pel-B, EHEB and Bonna-12) or the loss of function of the inhibitor of NFκB, namely IκB (L-428). This implies that many of these genes could be NFκB responsive. Twenty-four putative promoter regions were analyzed and NFκB-binding sites were identified in 12 of these. As expected, NFκB-binding sites were found in the CD40L receptor gene, *Bfl-1*, *BIRC3*, EBV-induced gene 3 (*EBI3*), and the genes for class I MHC-C and lymphotoxin *α*, as these have been previously characterized as NFκB responsive and were present in the initial NFκB-defined gene set. Interestingly, NFκB-binding sites were also found in six additional promoters for which accurate mapping of promoter transcription factor binding is not available (Figure 6a). All but four NFκB-binding sites conform precisely to the canonical consensus binding site (Figure 6b) [29,30] and of the variants with T at position 1, two genes, *lymphotoxin α *and *BIRC3 *are known to be NFκB responsive. Overall, this indicates that the six additional genes identified are likely to be NFκB responsive.

**Figure 7:**
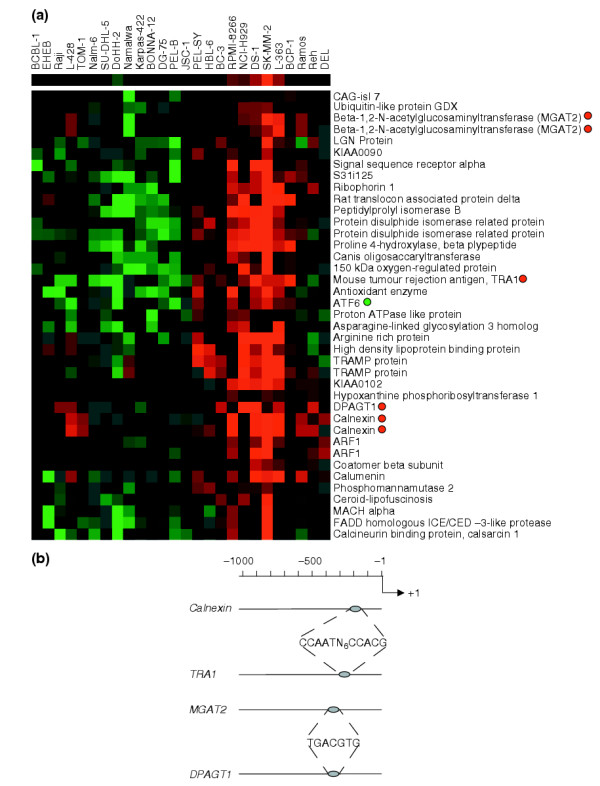
Genes involved in the ER/UPR. **(a) **Visualization of the ER/UPR consensus cluster using Cluster and Treeview. The *ATF6 *gene is indicated by a green circle. Gene names indicated by red circles represent those genes whose upstream 1,000-bp regions contain **(b) **the endoplasmic reticulum stress response element (ESRE), namely, *calnexin *and *TRA1*, or unfolded protein response element (UPRE) namely, *DPAGT1 *and *MGAT2*.

The consensus cluster associated with the ER/UPR functional group contained genes not previously associated with ER stress-induced upregulation. The gene-expression profiles were visualized and annotated as described for the NFκB functional group (Figure 7a). Annotation showed that of the 32 genes within the ER/UPR consensus cluster (23 defining the original functional group), 16% (5) were involved in calcium-ion binding within the ER and 13% (4) were involved with *N*-glycan biosynthesis. This functional group was overexpressed in cell lines of plasmablast or plasma-cell tumors, where physiological upregulation of the ER is required for cellular function. This process is controlled by two transcription factors, ATF6 and XBP1 [31]. The *ATF6 *transcript was present as a defining signature gene in the ER/UPR functional group. This suggests that ATF6 and XBP1 may be responsible for upregulation of the calcium-ion binding and *N*-glycan biosynthetic genes. Two responsive elements have been defined for ATF6 and XBP1 respectively, the ER stress-response element (ESRE), comprising the binding site CCAATN_9_CCACG and the unfolded protein response element (UPRE), comprising the binding site TGACGTG(G) [32]. ATF6 and XBP1 can bind to the CCACG region of ESRE in conjunction with the general transcription factor NF-Y/CB. XBP1 can bind to the UPRE, but ATF6 can only bind to the UPRE when expressed to high (possibly non-physiological) levels [33]. ESRE sites were identified in two of the five calcium-ion binding proteins, namely, calnexin and the tumor rejection antigen (gp96) 1(TRA1) (Figure 7b). Interestingly, XBP1 (UPRE) binding sites were identified in two of the *N*-glycan biosynthetic genes but no ESRE sites were found. This suggests that these two groups of genes are regulated through two distinct mechanisms by transcription factors ATF6 and XBP1 as a result of ER stress.

## Discussion

Grouping data into sets based on a consistent property is a common occurrence in biological analysis. This has recently increased in importance with the production of large microarray datasets. Implicit in the experimental rationale is the fact that patterns of coexpressed genes should be identifiable in a gene expression matrix and these can be linked to shared biological processes. However, different clustering algorithms are known to partition data into different groups [10-13]. We also observe a similar lack of cluster-method concordance using a *weighted-kappa *metric. This metric effectively scores how well different cluster method pairs assign the same genes to the same clusters. The *weighted-kappa *metric readily shows that, even for highly correlated gene-expression profiles present in the ACS dataset, no two clustering algorithms have complete agreement, although the global search methods such as SA seem to produce the most consistent results. Overall this emphasizes that no single analysis method will identify all patterns in the gene-expression data; therefore multiple analyses should be performed and compared [23].

We and others recently described the use of consensus clustering to improve confidence [34] or as a re-sampling method for microarray analysis [19]. It was suggested that a natural extension of Monti *et al*. was to use a meta-consensus across different clustering algorithms rather than to re-sample over the same algorithm. Our results represent this extension and confirm the validity of consensus clustering. We have developed both robust and consensus clustering, with these methods offering specific advantages over the use of individual clustering algorithms for microarray analysis. The robust clustering algorithm is useful for creating clusters of genes with high confidence and is extremely effective for reducing the dimensionality of large gene-expression datasets. However, robust clustering can be restrictive in discarding genes that do not have full agreement. Consensus clustering overcomes this problem, requiring a minimum-agreement parameter to generate clusters based on the combined results of a number of existing clustering methods. This strategy enables the effective identification of cluster groups that are of high reliability and cluster method independent.

The choice of clustering algorithms and parameter settings is a major stumbling block for all gene array cluster analysis. The effect of varying the parameters depends on the cluster method used. The performance of cluster methods has been extensively investigated [12]. The authors show that model-based methods and certain partitional methods, when used with optimal distance matrices, perform well on synthetic and real-world data. From our study, SA, an optimization method, also performs well as a clustering method. Therefore, the individual algorithms used as input to consensus clustering should ideally consist of representative algorithms from optimization (for example, SA), graph theoretical (for example, CAST), model-based (for example, MCLUST [12]) and partitional (for example, HC). Some methods (for example, CAST, SA and MCLUST) can determine the number of clusters directly from the input data. However, some other methods require the number of clusters to be specified as a parameter (for example, PAM and HC). In principle, methods such as CAST, SA and MCLUST can be used to determine this parameter for methods such as PAM and HC.

Consensus clusters are likely to contain gene subsets that are co-regulated by common transcriptional control networks or are coexpressed to participate in cellular processes that together manifest a global phenotype of the cell or tissue. In either case, these clusters are of high biological value. To facilitate further analysis it is useful to know which clusters are involved in a given biological process. By supplying a list of genes from a given biological process or network, the use of the normal approximation to the binomial distribution of these genes over all consensus clusters, allows the identification of clusters of high functional significance. Similar statistical assignment of gene function based on cluster analysis was performed by Wu *et al. *using a database of clusters [20]. To assign significance Wu *et al. *used the hypergeometric distribution. This distribution can be formally shown to asymptotically become the binomial distribution when the population size increases. Therefore, when used on large gene-expression datasets our methods are directly analogous to Wu *et al*. However, consensus clustering has the advantage over a database of clusters by producing low-probability clusters containing a significant percentage of known elements from functional groups.

Two functionally significant clusters, the NFκB-responsive cluster and the ER/UPR cluster, were investigated further here. Within the NFκB-responsive cluster 50% of the putative promoters of genes investigated had canonical NFκB-binding sites within 1,000 base pairs of the transcriptional start site, suggesting that they are NFκB responsive [29,30]. The majority of these genes had NFκB-binding sites within 500 bp of the transcriptional start sites, consistent with the location in other NFκB-responsive genes [35]. Of the remaining two genes with NFκB-binding sites greater than 800 bp from the transcription start site, one, *Bfl-2*, has been experimentally verified [36]. Analysis of the ER/UPR consensus cluster also provided information on gene regulatory elements, but more interestingly provides insights into the control and effect of the ERSR/UPR.

In cells, the presence of unfolded proteins in the ER is associated with induction of the ER/UPR. However, during the maturation of B-cells to antibody-secreting plasma cells, expansion of the ER to accommodate increased secretion of immunoglobulins is thought to be coupled to the final stages of plasma-cell maturation. The induction of the ER/UPR occurs via the coordinated activation of the transcription factors ATF6 and XBP1 [31,33]. ATF6 is normally maintained as an inactive, ER-resident, transmembrane protein that is cleaved, after translocating to the Golgi upon ER stress, by the site proteases S1P and S2P [37,38]. The cleaved transcriptionally active ATF6 is then free to translocate to the nucleus, where it can activate target genes such as XBP1 and the ER chaperon protein GRP78/BiP [39]. *XBP1 *mRNA is cleaved by the ER stress activated protein IRE1 to yield the transcriptionally active form of XBP1, inducing further genes of the UPR [32]. The activation of both the ATF6 and IRE1/XBP1 pathways results in enhanced transcription of ESRE-responsive genes; however, only XBP1 appears able to transactivate the UPRE. The identification of ESRE binding sites in the promoter regions of genes for calcium-ion binding protein and UPRE binding sites in the promoter regions of *N*-glycan biosynthesis genes suggests that these genes are differentially regulated by ATF6/XBP1 and XBP1 respectively.

The only known UPRE target gene is ER degradation-enhancing *α*-mannoside-like protein (EDEM), whose induction depends solely on IRE1/XBP1 activity [33]. Induction of the two UPRE-containing genes, UDP-GlcNAc:dolichol phosphate *N*-acetylglucosamine-1 phosphate transferase (*DPAGT1*) and UDP-GlcNAc:*α*-6-D-mannoside *β*-1-2-*N*-acetylglucosaminyltransferase II (*MGAT2*), which catalyze essential steps in the biosynthetic pathway of complex *N*-linked glycans, supporting a clear link between the dolichol pathway and the UPR [40]. In addition, the ER/UPR functional group suggests that *DPAGT1 *and *MGAT2 *expression is regulated solely by the IRE1/XBP1 pathway. Altogether, these results show that consensus clustering and gene functional group analysis provide a highly accurate way of mining gene-expression data for novel insights into different genes within the cluster.

Robust and consensus clustering provide a platform for more efficient microarray analysis pipelines. There is effectively no limit to the number of different clustering algorithms that can be used to feed into the consensus clusters, and each clustering algorithm could be run under different parameter sets to fully explore a microarray dataset [19]. In addition, different distance matrices could be used as input into the range of clustering algorithms. In each case the consensus clustering algorithm effectively acts as the collation and interpretation point for the different individual analysis methods. This environment is ideal for use in parallel processing computer farms and the GRID [41]. In such an environment, each node of the farm could perform a range of analyses with a subset of clustering algorithms, with the master node compiling the consensus results. This would greatly increase computational speed and allow a thorough, single data entry point, access to an extensive range of clustering methods.

All areas of functional genomics that produce high-dimensional datasets with inherent patterns will require data partitioning to allow interpretation. Consensus clustering in the context of statistically defined functional groups could allow a consistent analysis platform for such diverse data types.

## Materials and methods

### Clustering methods

We implemented and compared a representative sample of methods from the statistical, AI and data-mining communities. The methods used were average linkage HC, PAM, SA and CAST. As all the clustering techniques use correlation between variables, we used the Pearson's correlation coefficient, *r*, to measure the linear relationship between two variables, *x*_1 _and *x*_2_, where the variable can be either discrete or continuous [42]. HC and PAM methods were implemented using the statistical package R [22], while CAST and SA were implemented locally in C++.

HC is an agglomerative method that produces a hierarchical (binary) tree or dendrogram representing a nested set of data partitions. It has been applied successfully to many gene-expression datasets [43]. Sectioning a hierarchically clustered tree at a particular level leads to a partition with a number of disjointed groups, thereby yielding different clustering of the data. The tree was sectioned using the CUTTREE method, to yield 13 clusters for the ASC dataset and 40 for the B-cell dataset. The method PAM works by first selecting *m *out of *n *total objects that are the closest (according to a distance matrix) to the remaining (*n *- *m*) objects. The fitness of these medoids is calculated by placing the remaining (*n *- *m*) objects in a group according to the nearest medoid and summing the distances of the group members from this medoid. These *m *selected objects are the initial medoids. A *Swapping *procedure is then applied to reassort the objects until there is no improvement in the fitness of the medoids [3]. As with HC, PAM is set to search for 13 and 40 clusters. The choice of 13 clusters for the ASC data was determined by the number of repeated genes, whereas 40 clusters for the B-cell data was based on previous exploratory data analysis [21].

SA [6] is an iterative improvement search technique that starts with a random solution to a given problem, and then tries to increase its worth by a series of small changes in cluster membership. If such a small change is better than the previous solution, then further changes are made from this new point. However, if the new solution is worse than the old one, it is not discarded, but accepted with a certain probability. The measured worth of the SA clustering arrangement is based here on the EVM metric [44]. SA has recently been applied to the clustering of gene-expression data [45]. The performance related parameters for SA were set as follows: *θ*_0 _= 100, *c *= 0.99994 and number of iterations = 1,000,000.

CAST [9] is a heuristic algorithm that uses an affinity measure to determine whether variables are assigned to clusters. It requires a threshold parameter, which determines whether variables are assigned or moved to new clusters. Once CAST is complete, a clean-up operation is applied to ensure that the affinity of every variable to its cluster is greater than a user defined threshold. The only parameter CAST needed was the affinity level, which was set to 0.5 as recommended [9].

Methods such as CAST and SA require the differences/relationships between a pair of observations, *x*_1 _and *x*_2_, to be expressed as binary (*b*). As Pearson's correlation coefficient is bounded, it provides a good basis for defining a binary relationship between two variables as defined by:



where 0 <*α *≤ 0 is a constant and  is a floor function that returns the largest integer less than or equal to the real number *y*.

### Datasets for evaluation

Two datasets were used for evaluating the cluster methods. The first is a set of multiply repeated control element spots relating to the Amersham Score Card (ASC) probe set on the Human Genome Mapping Project Human Gen1 clone set array [46]. The ASC probes are present as a single row of 32 elements in each of the 24 array sub-grids. Of these elements, 13 gene probes consistently give signals above background in both the Cy5 and Cy3 channels. Therefore, each array has effectively 24 repeat measurements of 13 spots. After filtering for low signal-to-noise ratio (SNR) probes, a dataset of 30 arrays was examined by treating each positional repeat probe element across the 30 array set as an individual gene, which together with the remaining 23 same-gene probes per array, represents a highly correlated gene-expression profile. Therefore, we assume the repeated probes should cluster together; thus, this dataset becomes 308 genes/probe elements, which would cluster into 13 known groups, with each group having between 6 and 24 members after SNR filtering. In essence, the ASC data represent a semi-synthetic dataset for internal cluster method validation.

The second dataset consists of a series of 26 arrays (1,987 filtered genes) measuring gene-expression difference across a set of human B-cell lymphomas and leukemias [21]. The dataset is available via the URL indicated in Jenner et al. [21]. Each probe on the array detected a single gene transcript. This dataset contains a number of genes that correspond to known cellular functions, for example cell proliferation and NFκB response. The four clustering techniques described above were applied to both the datasets, with each method being set to find 13 clusters for the ASC and 40 clusters for the B-cell data.

### Synthetic datasets

Two synthetic datasets were generated using a vector autoregressive process (VAR) or a multivariate normal distribution (MVN). The two datasets contained 1,830 genes and 20 conditions (arrays). The VAR process of order *p *is a linear multivariate time series defined by



where *x*(*t*) is the *n*-dimensional vector of observations at time *t*, *A*_*i *_is the *n *× *n *autoregressive coefficient matrix at time lag *i*, and *ε*(*t*) is the zero mean *n*-dimensional noise vector at time *t *(drawn from a normal distribution). Therefore *x*(*t*) is a linear combination of the previous observations plus some random noise. For the synthetic dataset, each cluster was generated by a vector autoregressive model of order *p *= 1 and size *n *equal to the number of genes in the cluster.

For the MVN dataset, a vector of random variables *x* has a MVN distribution if every linear combination of that vector is also normal. Under such conditions we use the notation *x* ~ *N*(*μ*, Σ) to denote that *x* follows the MVN distribution, where *μ* is the mean vector and Σ is a positive definite matrix of covariance. The probability density function of *x* is given by



where |Σ| = det(Σ). For the synthetic dataset, each cluster was drawn from an MVN distribution with varying mean *μ* and covariance Σ.

### *Weighted-kappa *metric

To compare the resultant clusters for each method, a statistic known as *weighted-kappa *was used [18]. This metric rates agreement between the classification decisions made by two or more observers. In this case the two observers are the clustering methods. The classification from each observer for each unique pairing of variables (within the clusters) is divided into a 2 × 2 contingency table. Rows and columns within this table are indexed according to whether the two variables are in the same group or in different groups. The total number of comparisons, *N*, is defined in the following equation, where *Count*_*ij *_is the number of elements in the matrix cell indexed by (*i*,*j*),



and *n *is the number of variables (genes) in the clusters as this represents the number of unique variable pairings.

The *weighted-kappa *metric is calculated from the contigency table by:



where, *w*_*ij *_is the weights for each category comparison; *p*_*o*(*w*) _and *p*_*e*(*w*) _represent the observed weighted proportional agreement and the expected weighted proportional agreement; *Count*_*ij *_is the *i*th, *j*th element of the 2 × 2 contingency table; *N *is the sum of the elements within this table; *Row*(*i*) and *Col*(*i*) are the row and column totals for this table respectively and *K*_*w *_is the *weighted-kappa *value. The interpretation of *weighted-kappa *values indicates the strength of agreement between two observers (Table 1) is used to compare cluster method agreement in both datasets.

## Figures and Tables

**Table 1:** The *weighted-kappa *guideline

*Weighted-kappa*	Agreement strength
0.0 ≤ *K *≤ 0.2	Poor
0.2 <*K *≤ 0.4	Fair
0.4 <*K *≤ 0.6	Moderate
0.6 <*K *≤ 0.8	Good
0.8 <*K *≤ 1.0	Very good

**Table 2:** Robust clusters

Dataset	ASC*	B-cell
Number of robust clusters	24	154
% of variables assigned	79.2%	25%
Maximum robust cluster size	44	14
Minimum robust cluster size	2	2
Mean robust cluster size	10.2	3.2

*Amersham Score Card dataset.

**Table 3:** Multiple runs of the stochastic clustering methods

Method	Mean*	Min^†^	Max^†^	SD^†^
CAST	0.646	0.448	0.769	0.092
Simulated annealing (SA)	0.816	0.794	0.838	0.015
Consensus clustering (CC)	0.960	0.922	0.982	0.010

*Mean *weighted-kappa *scores; ^†^Min (minimum) and Max (maximum) and SD (standard deviation) of the *weighted-kappa *scores.

**Table 4:** Cluster partition *weighted-kappa *scores of two synthetic datasets

Dataset	HC	PAM	CAST	SA	CC
Vector autoregressive	0.505	0.700	0.537	0.614	0.725
Multivariate normal	0.697	0.605	0.591	0.667	0.729

HC, hierarchical clustering; PAM, partitioning around medoids; CAST, cluster affinity search technique; SA, simulated annealing; CC, consensus clustering.
